# The use of standardized management protocols for critically ill patients with non-traumatic subarachnoid hemorrhage: a protocol of a systematic review and meta-analysis

**DOI:** 10.1186/s13643-018-0716-7

**Published:** 2018-04-02

**Authors:** Shaurya Taran, Vatsal Trivedi, Jeffrey M. Singh, Shane W. English, Victoria A. McCredie

**Affiliations:** 1 Division of Internal Medicine, Department of Medicine, University of Toronto, Toronto, ON Canada; 2 Department of Anesthesiology and Pain Medicine, University of Ottawa, Ottawa, ON Canada; 3 Interdepartmental Division of Critical Care Medicine, Department of Medicine, University of Toronto, Toronto, ON Canada; 4 Division of Critical Care Medicine, Department of Medicine, University Health Network, Toronto, ON Canada; 5 Department of Medicine (Critical Care), The Ottawa Hospital, Ottawa, ON Canada; 6 Department of Medicine, University Health Network, 200 Elizabeth Street, Eaton Building 14-217, Toronto, ON M5G 2C4 Canada

**Keywords:** Subarachnoid hemorrhage, Standardized management protocols, Care pathway, Clinical algorithm, Critical care

## Abstract

**Background:**

Caring for patients with subarachnoid hemorrhage (SAH) presents unique challenges, due in part to the severity of the underlying insult, competing systemic injuries, and unpredictable clinical course. Even when management occurs in dedicated critical care settings, treatment uncertainty often persists, and morbidity and mortality from the condition remain high. Complex decisions in SAH care may be simplified with the use of standardized management protocols (SMPs). SMPs incorporate evidence-based guidelines into a practical framework for decision-making, thereby providing clinicians with an algorithm for organizing treatments. But despite these potential advantages, it is currently unknown whether SMPs may improve outcomes in the critical care of patients with SAH.

**Methods:**

We will conduct a systematic review of cohort studies and randomized control trials of adult patients with non-traumatic SAH who received care according to a standardized management protocol. Comprehensive search strategies will be developed for MEDLINE, EMBASE, WoS, CINAHL, and CENTRAL, to identify studies for review. The gray literature will be scanned for further eligible studies. Two reviewers will independently screen the material generated by the search to identify studies for inclusion. A standardized data extraction form will be used to collect information on study design, baseline characteristics, details of the management protocol employed, and primary and secondary outcomes. Where possible, meta-analyses with random-effects models will be used to calculate pooled estimates of effect sizes. Statistical heterogeneity will be evaluated with the *I*^2^ statistics, and risk of bias and reporting quality will be assessed independently and in duplicate with standardized scales.

**Discussion:**

We anticipate a significant degree of clinical heterogeneity in our review, as protocols will likely vary in their content, implementation, and ICU setting. We will aim to summarize the current literature in this domain to understand if SMPs, as a low-cost process-targeted intervention, improve outcomes for critically ill patients with SAH. Our review will additionally inform future research endeavors to improve the processes of care for this patient population.

**Systematic review registration:**

CRD42017069173

**Electronic supplementary material:**

The online version of this article (10.1186/s13643-018-0716-7) contains supplementary material, which is available to authorized users.

## Background

Subarachnoid hemorrhage (SAH) is a complicated disease entity associated with high mortality and morbidity. Despite advances in management and an improved understanding of its pathophysiology, in-hospital mortality remains around 20% [1] and long-term cognitive and functional outcomes are compromised in up to half of all survivors [2]. Costs of acute in-hospital treatment and outpatient care additionally represent a major burden on healthcare resources [3]. Owing to its difficult and often unpredictable course, the inpatient treatment of SAH is rarely straightforward, and downstream neurologic complications (rebleeding, delayed cerebral ischemia, elevated intra-cranial pressure, and seizures) add a further layer of complexity to its management. Systemic complications may also manifest through the disease course, adding to overall morbidity and mortality. Furthermore, practice pattern variation between physicians and institutions often persists despite the availability of many evidence-based clinical guidelines and consensus statements.

Recently, there has been considerable interest in the use of standardized management protocols (SMPs) to streamline medical care for complex, critically ill patients [4]. SMPs may facilitate communication, reduce cognitive load, and increase the adoption of evidence-based interventions. SMPs may also reduce treatment uncertainty and improve patient outcomes in the short and long term. This in turn may streamline the provision of limited healthcare resources, yielding more efficient care. The use of protocols has become standard practice in a variety of inpatient settings, with pre-printed order sets and care pathways being shown to improve patient outcomes for common admission diagnoses, such as congestive heart failure and recovery after hip surgery [5, 6]. In the intensive care unit (ICU), protocols have been studied in a variety of settings, including traumatic brain injury (TBI) [7], mechanical ventilation [8], and acute respiratory distress syndrome (ARDS) [9]. Protocol use in each of these conditions has been associated with varying degrees of benefit, compared with usual care.

Treating patients with SAH is a dynamic effort, requiring intensive care management and a high degree of interdisciplinary collaboration. Even where there is little evidence supporting individual treatment decisions, SMPs may reduce practice variability and allow clinicians to navigate therapeutic uncertainty in SAH. Furthermore, as many patients with SAH are cared for in large academic centers, SMPs may play an important role in standardizing clinical practice across rotating medical trainees. In this systematic review, we seek to assess the implementation and efficacy of SMPs in the critical care of SAH. Our primary objective is to determine whether standardized management protocols improve outcomes in adult patients with non-traumatic SAH, compared to those receiving usual treatment. If able, we additionally hope to synthesize reported outcomes in a meta-analysis to inform future research and quality improvement initiatives.

## Methods

### Design

A team of investigators including neuro-intensivists, internists, epidemiologists, and a biostatistician collaborated to develop the research question and study design. This systematic review will follow the guidelines set out in the Cochrane Handbook for Systematic Review and Meta-Analyses [10], and the protocol was registered in PROSPERO (https://www.crd.york.ac.uk/PROSPERO/). The research methodology presented in the final manuscript will adhere to the Preferred Reporting Items for Systematic Reviews and Meta-Analyses (PRISMA) guidelines [11].

### Information sources and search strategy

MEDLINE, EMBASE, Web of Science (WoS), the Cumulative Index to Nursing and Allied Health Literature (CINAHL), and the Cochrane Central Register of Controlled Trials (CENTRAL) will be systematically searched from their inception date to March 2017. A review of the gray literature will be performed to identify unpublished or ongoing studies using Google Scholar, https://clinicaltrials.gov and http://www.controlled-trials.com. This systematic review will also include a hand-search of the past 10 years of published abstracts from the conference proceedings of the World Federation of Neurological Surgeons and the European Federation of Neurological Societies, as these were not directly accessible through EMBASE. Conference abstracts from other relevant journals (the Neurocritical Care Society, the Society of Critical Care Medicine, the American Neurological Association, the American Thoracic Society, the Congress of Neurological Surgeons, the American Association of Neurological Surgeons, the American Academy of Neurology, the Canadian Neurological Sciences Federation, the European Neurological Society, the World Congress of Neurology, and the International Symposium on Intensive Care and Emergency Medicine) are indexed in EMBASE and will not be hand-searched. Before submission for publication, the search will be rerun through each database indicated above to account for newly reported findings. Authors of included studies will be contacted to clarify any unclear or unavailable information as necessary.

With the collaboration of a Health Information Specialist, the search strategy will be carefully developed to capture all studies of potential interest, using a combination of free text keywords and subject headings terms to yield the final search algorithm. Finally, the reference lists of each selected study will be scanned to identify any additional eligible studies for inclusion. A step-by-step breakdown of inclusions and exclusions will be provided in the final manuscript using the standard PRIMSA flow diagram. An example of our search strategy will be provided with the final manuscript.

### Eligibility criteria and study selection

We will apply the following eligibility criteria to identify studies for inclusion in this review: (1) randomized controlled trials and observational studies (including case series, prospective, and retrospective studies) (2) examining adult (age ≥ 18) patients with non-traumatic SAH in which (3) a standardized management protocol, care pathway, or algorithm was used. (4) The protocol or pathway must have been implemented during the acute admission period, and the publication must have (5) reported at least one of the primary outcomes (mortality, Glasgow Outcome Scale (GOS) or extended Glasgow Outcome Scale (GOSe)) or secondary outcomes of interest. The comparator of interest is a usual practice including before and after designs. Since we expect to find few studies examining the effectiveness of SMPs for non-traumatic SAH patients, we will include studies that did not assess outcomes against a comparator. In other words, studies with before and after designs without a comparison condition will be included in the review. Cluster RCTs will not be included since the unit of allocation of included studies must be the individual. No restrictions will be applied to type of treatment setting (i.e., neuro-ICU, medical/surgical ICU, or step-down ICU). Additionally, there will be no restrictions based on language or publication date.

Two reviewers will independently appraise all material generated by the search algorithm to identify studies for inclusion. Each reviewer will be blinded to the others’ appraisal of the literature. The first step of this process will involve independent screening by title and abstract to determine potential article eligibility. The second step will involve complete review of each study that has passed the initial screening. The final step will involve review of the reference list of each selected study to identify additional studies for inclusion. In cases of ambiguity, authors of the study in question will be contacted for clarification of uncertain information. This three-step approach will yield the final list of studies for analysis and data extraction. In the event of discrepancies in the final list generated by the two reviewers, a third reviewer will be consulted for arbitration. Reasons for exclusion at the second step of analysis will be presented in the final published manuscript.

### Data collection

Two independent reviewers will extract data from each study in the final list into a standardized pre-piloted data collection form (Additional file 1). We will collect data on (1) study design, including but not limited to study type, year of publication, inclusion and exclusion criteria, treatment setting (i.e., type of ICU), length of follow-up, sources of funding, and conflicts of interest; (2) baseline patient characteristics including age, sex, comorbidities, and mechanism of SAH (i.e., aneurysmal, arteriovenous malformation, perimesencephalic, dural arteriovenous fistula, arterial dissection); (3) type of medical or surgical treatment, including use of mechanical ventilation, tracheostomy, ventriculoperitoneal (VP) drains, and surgical clipping or endovascular coiling (in cases of aneurysmal SAH); (4) characteristics of the SMP, including whether it is presented as a tree diagram, flow chart, or pre-printed order set; or whether it is descriptive or numerical in nature; (5) healthcare provider adherence to the SMP (if reported); (6) and primary and secondary outcomes assessed in relation to the use of SMPs. The initial data abstraction form will be piloted on two studies to ensure robustness, with subsequent modifications for thoroughness if necessary. Duplicated studies will be included only once in the final analysis, with the most comprehensive article being represented.

### Assessment of methodological quality and risk of bias assessment

If our search identifies a randomized control trial (RCT) deemed eligible for inclusion, its risk of bias will be evaluated by two independent reviewers with the Cochrane Collaboration’s risk of bias tool [10]. However, we anticipate the majority of retrieved studies to be cohort designs and case series, and to this end, we will evaluate methodological quality with the Newcastle Ottawa Scale (NOS) [12]. The NOS is a validated eight-item checklist that assesses the quality of cohort and case series according to three broad domains. A star-system approach to grading allows for easy assessment of the variables of interest. The NOS will be modified to include the most important SAH prognostic variables (including age and SAH severity according to the World Federation of Neurological Societies (WFNS) classification, Hunt/Hess scale, or modified Fisher scale) under the comparability category. This will allow the reviewers to optimize the applicability of the scale to the SAH cohort studies. When considering comparability in the NOS, we will assess whether these important variables are adjusted for in the analysis. Summary reports on the quality of each represented study will be presented in graphical format in the final manuscript. The assessment of both risk of bias and quality will be undertaken independently by two reviewers.

### Assessing the quality of evidence

The Grading of Recommendations Assessment, Development and Evaluation (GRADE) framework will be used to assess the quality of evidence for each reported primary outcome [13]. This approach describes the level of confidence for which an estimate of effect is close to the value of interest. The GRADE assessment is based on the following criteria: risk of bias and study limitations, directness and consistency of results, precision, publication bias, magnitude of effect, dose-response gradient, and residual confounding. The level for the quality of evidence is summarized as high, moderate, low, or very low. If able, we will provide a final quality of evidence grade and strength of recommendation (strong or weak) in our review.

### Outcomes

Our primary outcome will be mortality at 6 months or greater. We will additionally assess neurologic outcome as defined by the Glasgow Outcome Scale (GOS), Functional Independence Measure (FIM), or the Disability Rating Scale (DRS), at hospital discharge and long-term follow-up. Studies reporting neurologic outcomes according to metrics other than the ones specified will also be considered, as long as these studies meet the rest of the eligibility criteria. Studies vary widely in how they report their outcomes; hence, we have chosen to be as inclusive as possible. Secondary outcomes of interest will include short-term mortality, defined as death within 21 days; length of stay in hospital; duration of mechanical ventilation; rates of adverse events and complications including ventilator associated pneumonia, CNS infection, seizure occurrence, delayed cerebral ischemia (DCI), radiographic vasospasm, and raised intracranial pressure (ICP).

### Statistical analysis and data synthesis

Data will be extracted from the standardized form and presented in a descriptive manner. Categorical data will be reported in proportions while continuous data will be presented as means with standard deviations or medians with ranges depending on the format used in the primary studies. SMPs will be classified as having a positive or negative effect on primary outcome, whenever possible, thus converting to a dichotomous variable. If SMP use leads to an improvement in one or more primary outcomes, that result will be classified as “positive,” whereas if the opposite is true, the result will be considered “negative.” We will use random-effects models (DerSimonian and Laird method) to calculate pooled estimates of effect sizes, if a meta-analysis of the pooled data is possible, using Review Manager 5.3.5 software (Cochrane Collaboration, Oxford, UK). Pooled continuous-effect measures will be expressed as mean differences (MD) and pooled dichotomous effect measures as risk ratios (RR), both with 95% confidence intervals (CI).

Heterogeneity between studies will be assessed using the *I*^2^ statistic. *I*^2^ will then be classified as the proportion of observed effects [14] or percentage of the variability in effect estimates that is due to heterogeneity rather than sampling error [10]. Interpretations regarding the significance of heterogeneity will be made as per standard characterization as negligible (< 40%), moderate (30–60%), substantial (50–90%), or considerable (75–100%) [10]. The *Tau*^2^ metric will be reported for random-effects models. Studies will be pooled by design, and meta-analysis will be carried out within study design. However, we will not report meta-analyses in the presence of high statistical heterogeneity. If studies involve more than two groups, we will compare the intervention group with the control group which is structurally closest to the intervention group. A narrative summary of the data will be undertaken if quantitative synthesis is not appropriate or possible.

Sensitivity and subgroup analyses will be performed, if permitted by the data available, to further characterize sources of heterogeneity and robustness of the results. We will perform a *z* test of interaction for all subgroup comparisons, which tests the null hypothesis that the treatment effects in each subgroup are the same. This analysis will be performed for the type of treatment setting (i.e., neuro-ICU vs non-neuro-ICU), severity of SAH, characteristics of the SMPs (e.g., descriptive vs. qualitative), timing of outcome assessment, and study risk of bias. Funnel plots will be constructed and visually inspected to assess for potential publication bias.

## Discussion

SAH is widely regarded as a resource-intensive disease that requires specialized treatment and expert management. Many patients go on to develop downstream neurologic dysfunction or systemic complications [15], which may prolong care and worsen outcomes. Surgical and endovascular treatment options for the initial hemorrhage have been clearly described and well studied in the literature [16, 17]. Guidelines also exist for the post-operative management of SAH patients [18–20], but there remains a high degree of practice pattern variation between clinicians and institutions in their overall approach to care.

SMPs have been proposed to introduce a level of uniformity into the treatment of medically complicated patients. Strong evidence supporting the use of SMPs currently exists for many common admission diagnoses, leading to their widespread use in many parts of the hospital. However, there is currently no summary evidence to indicate whether SMPs may improve outcomes in patients with SAH. This is certainly an area worth investigating since SMPs may represent a low-cost initiative for centers looking to improve their SAH-related processes of care. In this systematic review, we seek to collect and synthesize data from all studies looking at the use of SMPs in SAH, with the goal of informing practice recommendations. The methodology and study design will adhere to well-recognized standards of quality assurance. As a further step in our analysis, we will aim to assess the methodologic quality of included studies and examine their risk of bias. Our summary recommendations will allow for the assessment of patient-centered outcomes, identify evidence gaps, and determine key priorities for the organization of SAH care to guide future research initiatives.

Our effort to summarize the literature on this topic may be challenging due to the anticipated significant clinical and statistical heterogeneity between studies. Foreseeable limiting factors include differences in reported outcomes, types of protocols used, rates of clinician adherence to the SMPs, no comparator group, and patients’ baseline characteristics. Our final assessment will encompass both a summary recommendation of the literature as well as a global evaluation on the quality of reported studies. We plan to present the results of this systematic review at research conferences and aim to publish our findings in a peer-reviewed journal.

## Additional file

Additional file 1: Data extraction form for eligible studies. (DOCX 46 kb)

## Abbreviations

ARDS: Acute respiratory distress syndrome: a medical condition occurring in critically ill patients characterized by widespread inflammation in the lungs
DCI: Delayed cerebral ischemia: development of new focal neurological signs and/or deterioration in level of consciousness, lasting for more than 1 h, or the appearance of new infarctions on CT or MRI. The underlying pathophysiology is thought to be vasospasm after exclusion of other causes
DRS: Disability rating scale: a validated tool used to provide quantitative information regarding the progress of individuals with severe head injury. It is comprised of eight items in four categories: (i) level of consciousness, (ii) cognitive abilities, (iii) dependence on others, and (iv) employability
FIM: Functional independence measure: a tool used to evaluate the functional status of patients following a stroke, traumatic brain injury, spinal cord injury, or cancer
GOS: Glasgow Outcome Scale: a global scale for functional outcome that rates patient status into one of five categories: dead, vegetative state, severe disability, moderate disability or good recovery
GRADE: Grading of Recommendations Assessment, Development and Evaluation: a working group established in 2000 that aims to develop a common, sensible, and transparent approach to grading quality (or certainty) of evidence and strength of recommendations.
ICU: Intensive care unit: a department of the hospital where patients who are severely ill are kept under close observation
NOS: Newcastle Ottawa Scale: a validated eight-item checklist that assesses the quality of cohort and case series according to three broad domains
RCT: Randomized control trial: a study design that randomly assigns participants into an experimental group or a control group. As the study is conducted, the only expected difference between the control and experimental groups in an RCT is the outcome variable being studied
SAH: Subarachnoid hemorrhage: bleeding within the subarachnoid space resulting from a variety of mechanisms, including head trauma or aneurysmal rupture.
SMP: Standardized management protocol: an algorithm or care pathway used to guide clinical care and medical decision-making by healthcare personnel
TBI: Traumatic brain injury: a form of acquired brain injury that occurs when sudden trauma forces cause damage to the brain
