# Supplementary material for: The use of standardized management protocols for critically ill patients with non-traumatic subarachnoid hemorrhage: a protocol of a systematic review and meta-analysis
Source: Syst Rev. 2018 Apr 2;7:53. doi: 10.1186/s13643-018-0716-7 (PMC5880023; doi:10.1186/s13643-018-0716-7)
Supplement: Supplementary file 1 — Data extraction form for eligible studies. (DOCX 46 kb) [file 13643_2018_716_MOESM1_ESM.docx]

**Supplementary File 2: Data Extraction Form for Eligible Studies (SAH SMP)**

Reviewer: _____________________ Date of Extraction: ___________________

(dd/mm/yyyy)

**Article Identification:**

Title: _______________________________________________________________

_______________________________________________________________

_______________________________________________________________

_______________________________________________________________

First Author: _______________________________________________________________

Journal Name/

Reference Source: _____________________________________________________________________

Journal Article Published Abstract Conference Proceeding Other: ________

Year: _______ Volume: _______ Starting Page: _______

Full Publication: Yes No Comment: __________________________________

Language of Publication: English and / or _______________________________________

Source of sponsorship (Study): ________________________________________________________

Study – Country of Origin: ________________________________________________________

Number of Centres Participating: ________________________________________________________

Conflicts of Interest: ___________________________________________________________________

Comments: __________________________________________________________________________

**Verification of Eligibility:**

**Inclusion Criteria:** (verified by “tick” mark)

- Randomized Control Trial OR Observational study
- Patients ≥ 18 years of age
- Non-traumatic SAH
- Protocol-based care implemented during the acute admission period
- Reporting at least one of the outcomes of interest: (mortality, Glasgow Outcome Scare (GOS) or extended Glasgow Outcome Scale (GOSe)

**N.B. if study does not report outcomes, but only describes an institutional management protocol then please provide details below:**

________________________________________________________________________________

________________________________________________________________________________

________________________________________________________________________________

**Study Design:**

**Type of Study:**

- Randomized Control Trial
- Retrospective Cohort
- Prospective Cohort
- Cross-sectional study
- Case Control
- Case Series
- Descriptive study of protocol
- Other: _________________

**Inclusion Criteria:**

- - - 1. ___________________________________________________________________________
      2. ___________________________________________________________________________
      3. ___________________________________________________________________________
      4. ___________________________________________________________________________
      5. ___________________________________________________________________________
      6. ___________________________________________________________________________

**Exclusion Criteria:**

1. ___________________________________________________________________________
2. ___________________________________________________________________________
3. ___________________________________________________________________________
4. ___________________________________________________________________________
5. ___________________________________________________________________________
6. ___________________________________________________________________________

Type of ICU (if applicable): ________________________

Lead ICU MD (if applicable) : ________________________

Team members: Intensivist Neurologist Neurointensivist Neurosurgery Anesthesia Trauma Residents Nurse Nurse Educator/APN Not specified Other ________________­­­­­­­

**Protocol:**

**Protocol Characteristics:**

- Interprofessional derived
- Pre-securing of aneurysm driven
- Post-securing of aneurysm driven
- ICP/CPP driven
- DCI/vasospasm driven
- General NeuroICU driven
- MD care driven
- Nursing care driven
- Guideline Based, specify: __________________
- Descriptive (ie: no diagram)
- Admission orders
- Flow diagram

How was the protocol developed: _____________________________________________________________

(e.g. Consensus, Single MD, interprofessional, Delphi method, adapted from another institution)

Adherence to protocol (if reported): ­___________________________________________________________

Control group present

Management for control group: ______________________________________________________________

**Protocol Specifics:**

| **Variable** | **Intervention** | **Details** |  |
| --- | --- | --- | --- |
| General NeuroICU Management Protocol | Fluid balance  -Daily  -Q_____H  -Replacement strategy  -Goal-directed fluid management with transpulm. hemodynamic monitoring |  |  |
|  |  |  |  |
|  |  |  |  |
|  |  |  |  |
|  | Na balance |  |  |
|  | Hydrocephalus/IVH  -tpa via EVD  -drainage of CSF |  |  |
|  |  |  |  |
|  |  |  |  |
|  | Sedation management  -Sedation target/score:  -Agents used:  -Range of doses: |  |  |
|  |  |  |  |
|  |  |  |  |
|  |  |  |  |
|  | SBP/MAP targets |  |  |
|  | Pre-coiling/clipping  Post-coiling/clipping |  |  |
|  |  |  |  |
|  |  |  |  |
|  | Mechanical ventilation |  |  |
|  | Lung protective strategies |  |  |
|  | PEEP range |  |  |
|  | SpO2 targets  PaO2 targets  FiO2 range |  |  |
|  |  |  |  |
|  |  |  |  |
|  | PaCO2 targets |  |  |
|  | Other |  |  |
| ICP/CPP-Based Protocol | ICP target/range  CPP target/range |  |  |
|  |  |  |  |
|  | Intraparenchymal Monitor |  |  |
|  | External ventricular drain |  |  |
|  | Imaging |  |  |
|  | CSF drainage |  |  |
|  | Sedation/analgesia |  |  |
|  | Hypertonic Saline  % used  bolus dose  target |  |  |
|  |  |  |  |
|  |  |  |  |
|  | Mannitol  % used  bolus dose  target |  |  |
|  |  |  |  |
|  |  |  |  |
|  | Vasopressor Use |  |  |
|  | Type |  |  |
|  | Cooling |  |  |
|  | PaCO2 |  |  |
|  | Other |  |  |
| DCI/Vasospasm-Based Protocol  DEFINITION OF VASOSPASM:  DEFINITION OF DCI: | Mechanical ventilation |  |  |
|  | Imaging/monitoring |  |  |
|  | Transcranial Doppler |  |  |
|  | CT (plain CT or CTA or CTP) |  |  |
|  | Other (CEEG) |  |  |
|  | SBP target:  SBP Range:  MAP target:  MAP Range:  CVP Range:  PCWP Range:  Hematocrit Target: |  |  |
|  |  |  |  |
|  | Vasopressor Use: |  |  |
|  | Type |  |  |
|  | Fluid boluses |  |  |
|  | Inotropes |  |  |
|  | Nimodipine  -duration  -route (iv/enteral/intraventricular)  -reduced doses/hold for low BP |  |  |
|  |  |  |  |
|  |  |  |  |
|  |  |  |  |
|  | Other |  |  |
| ADDITIONAL ELEMENTS: |  | |  |
| Nutrition |  | |  |
| Tracheostomy |  | |  |
| Mechanical Ventilation |  | |  |
| PEG |  | |  |
| Hypothermia |  | |  |
| Barbiturate coma |  | |  |
| Multimodal neuromonitoring |  | |  |
| CEEG |  | |  |

Comments/Clarification: ____________________________________________________________

_________________________________________________________________________________

_________________________________________________________________________________

**Stated Outcomes:**

| **Outcome** | | **Primary** | **Secondary** | |
| --- | --- | --- | --- | --- |
| 1 |  |  |  |  |
| 2 |  |  |  |  |
| 3 |  |  |  |  |
| 4 |  |  |  |  |
| 5 |  |  |  |  |

**Screening and Enrollment:**

Total patients enrolled: ________ Control pts: ________ Intervention pts: ________

Timing of enrolment (mm/yy): Control pts: ________ Intervention pts: ________

Consecutive patients: Yes No

Description of why not: _______________________________________________________________________________________________

Participant identification:

- ICU database
- Prospective enrolment
- Other: __________________
- Hospital database
- Medical records coding

**Baseline Characteristics:**

|  | **Control** | **Intervention** |
| --- | --- | --- |
| **Demographics:** | | |
| Age (SD) |  |  |
| Sex (%) |  |  |
| APACHE (SD) |  |  |
| SOFA/MODS/SAPS |  |  |
| Admission GCS (SD) |  |  |
| Post-op admission (%) |  |  |
| Transferred from another institution (%) |  |  |
| **SAH:** | | |
| Mechanism: (%)   1. Aneurysmal 2. Arteriovenous malformation 3. Perimesencephalic 4. Dural arteriovenous fistula 5. Arterial dissection 6. Other |  |  |
|  |  |  |
|  |  |  |
|  |  |  |
|  |  |  |
|  |  |  |
|  |  |  |
| Severity: (%)   1. WFNS 2. Hunt/Hess 3. Modified Fisher |  |  |
|  |  |  |
|  |  |  |
|  |  |  |
| Treatment: (%)   1. Surgical clipping 2. Endovascular coiling |  |  |
|  |  |  |
|  |  |  |
| Location of aneurysm (%)  -MCA  -A comm  -P comm  -ACA  -PCA  -Other |  |  |
|  |  |  |
|  |  |  |
|  |  |  |
|  |  |  |
|  |  |  |
|  |  |  |
| Time to securing of aneurysm (clipping OR coiling) (IQR) |  |  |
| **Baseline Hemodynamics: (on admission** **/ day 1** **/ median 7 days)** | | |
| ICP |  |  |
| CPP |  |  |
| SpO2 |  |  |
| PaCO2 |  |  |
| BP |  |  |
| HR |  |  |
| Temperature |  |  |
| GCS |  |  |
| CVP |  |  |
| SvO2 |  |  |
| Other |  |  |

**Outcomes:**

|  | **Control** | **Intervention** |
| --- | --- | --- |
| **Mortality:** | | |
| Death (%) |  |  |
| Withdrawal of Life Sustaining Treatments (%) |  |  |
| Non SAH-related (%) |  |  |
| Time to death (IQR) |  |  |
| **Morbidity:** | | |
| ICU LOS |  |  |
| Hospital LOS |  |  |
| Ventilator days |  |  |
| Day of tracheostomy insertion |  |  |
| Initiation of feeds |  |  |
| Day of PEG insertion |  |  |
| GOS |  |  |
| Discharge status |  |  |
| **Physiologic/Adverse Events:** | | |
| Rebleed |  |  |
| Hydrocephalus |  |  |
| Hyponatremia |  |  |
| OR (ie: craniectomy) |  |  |
| Seizure |  |  |
| Raised ICP (cerebral edema) |  |  |
| DCI/vasospasm |  |  |
| Pneumonia (VAP/HAP) |  |  |
| CNS infection |  |  |
| Short term mortality (death by 21 days) |  |  |
| Respiratory failure |  |  |
| Heart failure |  |  |
| Other (Specify) |  |  |
| **Resources:** | | |
| Health Care Costs |  |  |

**NEWCASTLE - OTTAWA QUALITY ASSESSMENT SCALE**

**COHORT STUDIES**

Note: A study can be awarded a maximum of one star for each numbered item within the Selection and Outcome categories. A maximum of two stars can be given for Comparability

**Selection** (tick one box in each section)

1) Representativeness of the intervention cohort

- Truly representative of the non-traumatic SAH population **🟑**
- Somewhat representative of the non-traumatic SAH population **🟑**
- Selected group of patients
- No description of the derivation of the cohort

2) Selection of the non-intervention cohort

- Drawn from the same community as the intervention cohort **🟑**
- Drawn from a different source
- No description of the derivation of the non-intervention cohort

3) Ascertainment of intervention

- Secure record (e.g. health records) **🟑**
- Structured interview **🟑**
- Written self-report
- Other/no description

4) Demonstration that outcome of interest was not present at start of study

- Yes **🟑**
- No

**Comparability** (tick one or both boxes as appropriate)

1) Comparability of cohorts on the basis of the design or analysis

- Study controls for Grade of SAH (WFNS, Hunt/Hess, Fisher, or modified Fisher) **🟑**
- Study controls for age **🟑**

**Outcome** (tick one box in each section)

1) Assessment of outcome

- Independent blind assessment **🟑**
- Record linkage **🟑**
- Self-report
- Other/no description

2) Was follow-up long enough for outcomes to occur

- Yes, if median duration of follow-up was 6 months or greater **🟑**
- No, if median duration of follow-up was less than 6 months

3) Adequacy of follow up of cohorts

- Complete follow up: all subjects accounted for **🟑**
- Subjects lost to follow up unlikely to introduce bias: number lost less than or equal to 20%, or description of those lost suggesting no different from those followed **🟑**
- Follow up rate of less than 80% and no description provided of those lost
- No statement

**TOTAL SCORE: /10**
